# Success and complications in lumbar punctures of pediatric patients with leukemia: a study protocol for a randomized clinical crossover trial of a bioimpedance needle system versus conventional procedure

**DOI:** 10.1186/s13063-023-07498-4

**Published:** 2023-07-21

**Authors:** Harri Sievänen, Juho Kari, Henri Aarnivala, Stefan Becker, Anu Huurre, Satu Långström, Sauli Palmu

**Affiliations:** 1 Injeq Plc, Tampere, Finland; 2 Department of Pediatric Hematology and Oncology, Oulu University Hospital, Oulu, Finland; 3 Department of Pediatric Hematology and Oncology, Kuopio University Hospital, Kuopio, Finland; 4 Department of Pediatric and Adolescent Hematology and Oncology, Turku University Hospital, Turku, Finland; 5 Department of Pediatric Hematology, Oncology and Stem Cell Transplantation, New Children’s Hospital, Helsinki University Hospital, Helsinki, Finland; 6 Tampere Center for Child, Adolescent and Maternal Health Research, Faculty of Medicine and Health Technology, Tampere University and Tampere University Hospital, Tampere, Finland

**Keywords:** Acute lymphoblastic leukemia, Lumbar puncture, Medical devices, Spinal needle, Traumatic puncture

## Abstract

**Background:**

Acute lymphoblastic leukemia (ALL) is the most common malignancy diagnosed in children. At present, the long-term survival from pediatric ALL is well over 90%. However, the probability of event-free survival is reduced if the lumbar puncture (LP) procedures at the beginning of the patient’s intrathecal therapy cause blood leakage into the spinal canal and blast cells contaminate the cerebrospinal fluid. According to the literature, such traumatic LP procedures concern one out of five pediatric patients with ALL.

Recently, a novel medical device measuring the tissue bioimpedance at the tip of a spinal needle was found feasible in pediatric patients with ALL. The LP procedure was successful at the first attempt in 80% of procedures, and the incidence of traumatic LPs was then 11%. The purpose of the present study is to compare the bioimpedance spinal needle system with the standard clinical practice resting on a conventional spinal needle and investigate its efficacy in clinical practice.

**Methods:**

The study is a multicenter, randomized, two-arm crossover noninferiority trial of pediatric hemato-oncology patients that will be conducted within the usual clinical workflow. Patients’ LP procedures will be performed alternately either with the IQ-Tip system (study arm A) or a conventional Quincke-type 22G spinal needle (study arm B). For each enrolled patient, the order of procedures is randomly assigned either as ABAB or BABA. The total number of LP procedures will be at least 300, and the number of procedures per patient between two and four. After each study LP procedure, the performance will be recorded immediately, and 1-week diary-based and 4-week record-based follow-ups on symptoms, complications, and adverse events will be conducted thereafter. The main outcomes are the incidence of traumatic LP, first puncture success rate, and incidence of post-dural puncture headache.

**Discussion:**

The present study will provide sound scientific evidence on the clinical benefit, performance, and safety of the novel bioimpedance spinal needle compared with the standard clinical practice of using conventional spinal needles in the LP procedures of pediatric patients with leukemia.

**Trial registration:**

ISRCTN ISRCTN16161453. Registered on 8 July 2022.

## Background

Acute lymphoblastic leukemia (ALL) is the most common malignancy diagnosed in children showing the peak incidence around 3–5 years of age [1]. The incidence of ALL is about 20–40 cases per million children and adolescents aged under 18 years old, depending on the geographic region (2). In developed countries, the improved survival from ALL has been a real success story during the last few decades, and at present, the long-term survival from ALL is well over 90% [1, 2].

Intrathecal chemotherapy administered by lumbar puncture (LP) is an essential part of ALL treatment. Besides the intrathecal therapy, the collected samples of cerebrospinal fluid (CSF) provide important information for the diagnosis, risk assessment, and choice of the patient’s treatment path as well as for the follow-up. Depending on the personalized risk classification, the total number of LP procedures may vary from five to more than 20 procedures during the roughly 2-year treatment period of ALL [3]. Regarding the patient’s optimal treatment and recovery from ALL, the successful and smooth conduct of the LP procedure throughout the treatment is of paramount importance.

The patient’s event-free survival reduces if the LP procedures at the beginning of the patient’s intrathecal therapy are traumatic and blast cells contaminate CSF because of blood leakage caused by a needle trauma [4–10]. Traumatic LPs (TLP), defined as a concentration of red blood cells in the CSF sample above a specified threshold, are frequent in clinical practice. In hemato-oncology, the threshold for TLP is at least 10 erythrocytes/μL of CSF. Compiling the reported data from almost 22,400 LP procedures of pediatric oncology patients [4–14], the mean incidence of TLP is about 20%, while the study-specific incidences of TLP vary from 1% [8] to 37% [13]. Without blood leakage into the subarachnoid space, there should not be any erythrocytes in CSF.

In pediatric patients, a higher number of attempted punctures during a single LP procedure is associated with a doubled risk of TLP [15]. However, one cannot presume constant success at the first attempt. In pediatric patients with ALL, the mean first puncture success rate of LP with a single skin penetration is good to excellent varying from 70% up to 95% [16–19]. In adults with leukemia, high success at the first attempt is associated with a roughly halved incidence of TLP [20], whereas in pediatric patients with ALL, the probability of TLP was about fourfold if the first attempt was not successful [19]. High success at the first attempt likely contributes also to a low incidence of post-dural puncture headache (PDPH) [17], which is the most considerable adverse event after an LP procedure. In children with ALL, however, the incidence of PDPH is yet relatively low varying around 10% [15–19].

Experience and training contribute to an improved performance in LP [17, 18]. The success of the LP procedure can also be improved by image guidance either before inserting the spinal needle into the body or in real-time during the procedure. According to recent meta-analyses [21–23], ultrasound guidance is associated with a halved incidence of TLPs, an improved success rate of LP procedures, and a reduced incidence of failed procedures compared to the traditional palpation-based procedures. Particularly patients with high body mass index (BMI), anatomic abnormalities, young age, or a history of many failed attempts may be referred to ultrasound imaging before or during the LP procedure [21, 24].

While the evidence for the clinical utility of ultrasound imaging in performing LP is convincing [21–23], the true real-time ultrasound guidance requires a physician’s one hand to use the ultrasonic probe and the other to advance the spinal needle, let alone the need for bedside imaging equipment, and provider’s adequate proficiency to employ ultrasound imaging and interpret the images in real-time. These requirements may modify the standard provision of LP the physicians who are accustomed to performing. Thus, a needle guidance method not requiring specific expertise and simultaneous operation of the imaging equipment but still providing real-time feedback on the needle tip location would be a valuable option.

Recently, clinical studies of adults [25], neonates and infants [26], and pediatric patients with ALL [19] showed the feasibility and safety of a novel bioimpedance-based spinal needle system in LP procedures. This system, called the IQ-Tip system, measures bioimpedance at the tip of the spinal needle with 15 different frequencies and classifies the bioimpedance data 200 times in a second. This means that the tissue classification is virtually real-time (performed at every 5th ms) and the spatial sensitivity is high (~ 1 mm^3^). The system gives an audio-visual alarm when the needle tip reaches CSF in the subarachnoid space and indicates the correct location for CSF sampling or administration of intrathecal therapy. In our recent single-arm study of pediatric patients with ALL, the first attempt was successful in 80% of procedures, and the incidence of TLP was 17%. Further, neither the success rate nor the incidence of TLP was associated with the provider’s experience or the patient’s overweight [19].

Given the promising real-world clinical experience, we hypothesize that the performance of the IQ-Tip system is at least comparable to the conventional spinal needle regarding the incidence of TLP in LP procedures of pediatric hemato-oncology patients. The primary objective of the present trial is to demonstrate that the observed incidence of TLP with the IQ-Tip system will indicate either noninferiority or not only noninferiority but also superiority compared to the conventional spinal needle, which is the current standard clinical method. The secondary objectives are to compare the first puncture success rate and the incidence of complications, particularly the incidence of PDPH, in the LP procedures performed with the IQ-Tip system and conventional spinal needle. No formal hypotheses are set for the secondary outcomes.

## Materials and methods

### Trial design and setting

The present study (acronym IQ-LP-04) is a multicenter, randomized, two-arm crossover noninferiority trial of pediatric hemato-oncology patients. Since the patients act as their controls in the cross-over design, the potential bias due to variability between the patient characteristics is effectively controlled for. The two study arms refer to performing the patient’s LP procedures alternately using the IQ-Tip system (study arm A) or a conventional spinal needle (study arm B). The arm of the first study procedure will be randomly assigned, while the number of procedures per patient will be between two and four. Thus, the two possible randomly assigned sequences of the study LP procedures are ABAB and BABA.

All study LP procedures are part of patients’ preplanned LP procedures without additional procedures or laboratory tests. Only the data needed for the management of patients’ malignant diseases will be collected as part of routine therapy and evaluation, independent of the present trial. Biological specimens for genetic or molecular analyses will not be collected. If the patient’s treatment or medical condition requires concomitant care, modification, or other interventions during the study, they will be permitted. The crucial point for the study is that the allocated spinal needles are used in the study LP procedures as intended. Patients’ treatments and care will continue as planned after completing the trial or withdrawing from the study due to any reason. There are no specific plans for promoting participant retention during the study. The pragmatic nature of the present trial is expected to promote complete follow-up and acquisition of data.

**Fig. 1:**
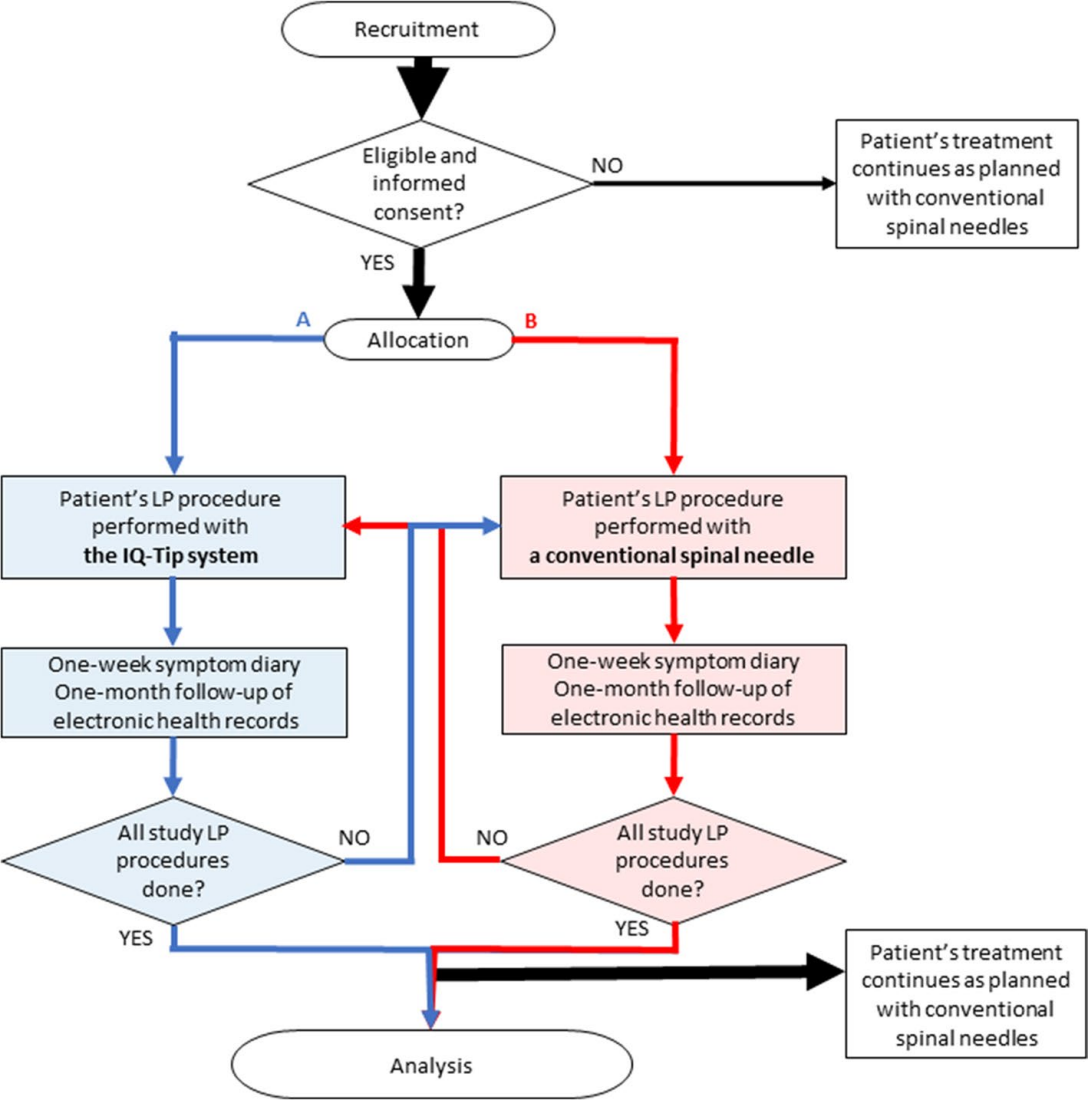
The flow chart of the study LP procedures

After each study LP procedure, there will be a 1-week follow-up using a symptom diary and a 4-week follow-up of hospital electronic health records. The flow chart of the study LP procedures is shown in Fig. 1, whereas the schedule of enrolment, study LP procedures, and assessments during the post-allocation period are outlined in Table 1. This protocol has been written according to the SPIRIT reporting guidelines [27] and verified against the SPIRIT checklist.

**Table 1:** Schedule of enrolment, interventions, and assessments (only those assessments pertinent to the efficacy analysis are presented) during the post-allocation period of the trial

Study period	Enrolment		Post-allocation												Close-out
**Time point**	*t*_−1_	*t*_0_	*t*_1_	*t*_1_ + 1wk	*t*_1_ + 4wk	*t*_2_	*t*_2_ + 1wk	*t*_2_ + 4wk	*t*_3_	*t*_3_ + 1wk	*t*_3_ + 4wk	*t*_4_	*t*_4_ + 1wk	*t*_4_ + 4wk	*t*_x_
**Enrolment**															
Eligibility assessment	x														
Informed consent	x														Archived
Random allocation		x													
**Interventions**															
LP procedure sequence ABAB			A			B			A			B			Tx continues as planned
LP procedure sequence BABA			B			A			B			A			
**Assessments**															
Baseline variables															
Age		x													
Height		x													
Weight		x													
Sex		x													
Main outcome variables															CRFs archived
Incidence of TLP			x			x			x			x			
First puncture success rate			x			x			x			x			Pseudonymized copies of CRF are delivered to the sponsor
Incidence of PDPH				x			x			x			x		
Other outcome variables															CRFs archived
Peripheral blood analysis			x			x			x			x			
CSF analysis			x			x			x			x			Pseudonymized copies of CRF are delivered to the sponsor
Number of LP attempts			x			x			x			x			
Number of failed procedures			x			x			x			x			
Symptoms, complications, and adverse events				x	x		x	x		x	x		x	x	

The study will be conducted in pediatric hemato-oncology departments of five Finnish university hospitals located in Helsinki, Kuopio, Oulu, Tampere, and Turku. These hospitals provide tertiary care for the entire Finnish population of about 5.6 million people. They are also responsible for the medical care of all childhood cancer patients living in their catchment areas.

The participating hemato-oncology departments are called the investigational sites, where the local principal investigators are responsible for conducting the study. All study LP procedures will be conducted within the daily clinical routine of the investigational sites. As the study procedures are part of the patient’s planned therapy for malignant disease, they will be covered by the hospital’s liability insurance. The study sponsor, a Tampere-based manufacturer of the IQ-Tip system (www.injeq.com), has liability insurance for covering the serious adverse events or harms affecting the participant that will be attributed to the IQ-Tip system.

### Ethical and regulatory considerations

The study protocol has been approved by the Regional Ethics Committee of the Expert Responsibility Area of Tampere University Hospital, Finland (date 10/05/2022, R22039L). The National Competent Authority (Fimea, Helsinki) evaluated and approved (date 04/07/2022) the study as per the national legislation. The trial will be conducted according to Helsinki Declaration and Good Clinical Practice (ISO 14155:2020).

A need to change some procedures in the study protocol may arise during the study. Should this happen, the principal investigator and the sponsor representatives will scrutinize the need and decide whether the protocol amendments are justified and necessary. If so, Ethics Committee and National Competent Authority will be notified about these amendments for approval before their implementation in the protocol.

### Registration

The trial has been registered on ISRCTN registry (date 08/07/2022, ISRCTN16161453). All items presented in the trial registration data set are also addressed within this protocol article.

### Recruitment and consent

Local study nurses or investigators will recruit participants in this trial among pediatric hemato-oncology patients of the investigational sites. All patients whose malignant disease requires multiple LP procedures in diagnostics or therapy are potentially eligible for the study. After having received written and oral information about the trial, the patient and/or parents may express their willingness to participate, sign the written informed consent, and give it to the recruiting person.

The consent applies to the use of all collected data in a pseudonymized form in scientific research, product development, and communication with authorities in potential safety and regulatory issues. Except for those mentioned in this protocol, there are no plans for ancillary studies.

The original participant’s trial information leaflet and the informed consent form, approved by the ethical committee and competent authority, are in Finnish and thus not given as supplementary material. In short, the leaflet describes the purpose and contents of the study; benefits and risks related to the study; principles of data protection, processing, and handling; voluntariness; statements of existing liability insurances and lack of incentives; and dissemination plans for the trial results.

A patient will be eligible for the trial, if:

- He/she is older than 1 year but no more than 18 years at the beginning of the study.
- His/her diagnosis or treatment plan requires multiple LP procedures for collecting CSF samples and injecting intrathecal therapy.
- The planned LP procedures will be performed with 22G Quincke-type spinal needles
- There are at least two LP procedures left in the patient’s treatment protocol.
- Both the parent(s) and the patient, depending on the patient’s age, give a signed informed consent before the first study LP procedure.

A patient will be excluded from the trial if:

- The parent(s) and/or the patient refuse to participate in the trial or the parent(s) and/or the patient are considered unable to give an informed consent.
- The provider considers the longest available spinal needle too short to perform the procedure safely.

An LP procedure of an otherwise eligible patient will be excluded from the study if:

- There is any temporary contraindication to LP, including but not limited to skin infection around the puncture area, unstable hemodynamics, bleeding tendency and increased intracranial pressure.

The final decision to include or exclude the patient or a single LP procedure (e.g., the initial procedure of the patient with ALL) will be up to the clinical judgement of the provider.

### Randomization and allocation

The spinal needle that will be used in the patient’s first study LP procedure, either the IQ-Tip system (study arm A) or a conventional spinal needle (study arm B), was determined by block randomization before recruiting patients. Block randomization ensures an approximately even number of both sequences of study LP procedures (ABAB and BABA) in random order while eliminating the bias that may arise from a fixed order of LP procedures.

With a dedicated Matlab script, the sponsor has generated randomization lists for each investigational site beforehand (date 30/09/2022). The sponsor will also prepare and deliver enough sequentially numbered sealed envelopes containing the randomly assigned sequences (either ABAB or BABA) to the investigational sites. During the trial, the person who will perform the statistical analyses will not meet the participants nor be involved anyhow in collecting data or filling their case report forms (CRF).

Upon enrolling a new patient to the study, the next available envelope in the order will be opened to reveal the sequence of study LP procedures for the given patient. This sequence determines the choice of a specific Participant’s File, a binder that contains color-coded CRFs and other relevant documents for collecting the data from the four study LP procedures. The running number of the envelope together with the hospital-specific three-letter prefix will determine the patient’s pseudonym (ID code).

### Procedures

The study LP procedures will be performed either with the IQ-Tip system (study arm A) or the conventional spinal needle (study arm B) according to the patient’s randomly allocated sequence. The personnel of the investigational sites will perform the study LP procedures within the usual clinical workflow. As a standard procedure, the LP procedures of pediatric patients with leukemia are performed under general anesthesia in the lateral decubitus position [19]. Because of general anesthesia, the patient is blinded to the allocated spinal needle. As both study needles are similar, the need for unblinding is deemed irrelevant.

Before starting the study, the sponsor’s representatives will give an overview of the Instructions for Use and a practical training session on the use of the IQ-Tip system to the local principal investigators (HA, SB, AH, SL, and SP), other physicians, and study nurses at the investigational sites. The attendees will also have the possibility to try the system on a lumbar phantom (Blue Phantom BPLP2201, CAE Healthcare, FL, USA). Later, the local principal investigators will be responsible for the training of new users of the system.

The above-described short training was employed in our previous single-arm study [19]. Immediately after that study, the usability of the IQ-Tip system was assessed with System Usability Scale (SUS) [28, 29]. In short, all 26 physicians who had performed at least one study LP procedure with the IQ-Tip system received an electronic SUS form, 20 of them (77%) responded anonymously. The mean SUS score was 75 (SD 13), which indicates good clinical usability and acceptability for the system.

#### The LP procedure A

Study procedure A will be performed with the IQ-Tip system (Injeq Plc, Tampere, Finland), which is a CE-marked medical device as per Medical Device Regulations (MDR EU 2017/745) approved for any clinically indicated LP procedure. The system comprises either a 40-mm, 65-mm, or 90-mm-long 22G Quincke-type spinal needle (IQ-Tip spinal needle), a bioimpedance analyzer, and a thin, flexible coaxial cable for connecting the needle to the analyzer. The IQ-Tip spinal needle is identical to the conventional Quincke-type 22G spinal needle with a stylet, except the stylet is configured as a bioimpedance electrode and it has a cable connector at the handle end. The analyzer has an internal memory, where the raw bioimpedance data collected at a 200-Hz sampling rate will be stored for further post-hoc analysis.

Performing the LP procedure with the bioimpedance needle is basically like that with the conventional spinal needle. Also, the removal and reinsertion of the stylet do not differ from the conventional spinal needle. Only the cable at the handle end is the major difference.

When performing the LP procedure with the IQ-Tip system and perceiving the CSF detection alarm, the provider can either remove the stylet and check for CSF flow through the needle or continue the puncture upon clinical judgment on the location of the needle tip. Similarly, irrespective of the alarm, the provider can remove the stylet and check for CSF flow whenever considered necessary.

When the needle tip is correctly in the subarachnoid space verified by CSF flow, the provider will take the CSF sample and deliver the intrathecal therapy according to the patient’s treatment protocol.

Right after performing the study LP procedure with the IQ-Tip system, the provider will evaluate the performance and record the results concerning a successful procedure at the first attempt, number of attempts needed for a successful procedure, number of stylet removals in the successful procedure, failure to perform the procedure, and the accuracy of perceived alarms on the patient’s CRF. Also, patients’ immediate complications and device deficiencies, if any, will be recorded on CRF.

#### The LP procedure B

Study LP procedure B will be performed with a conventional 22G Quincke-type spinal needle according to the standard clinical practices of the investigational sites.

When performing the LP procedure with the conventional needle, the provider can, upon clinical judgment on the location of the needle tip, remove the stylet and check for CSF flow through the needle, or continue the puncture.

When the needle tip is correctly in the subarachnoid space verified by CSF flow, the provider will take the CSF sample and deliver the intrathecal therapy according to the patient’s treatment protocol.

Right after performing the study LP procedure with the conventional needle, the provider will evaluate the performance and record the results concerning successful procedure at the first attempt, total number of attempts needed for a successful procedure, number of stylet removals in the successful procedure, and failure to perform the procedure on the patient’s CRF. Also, patients’ immediate complications and device deficiencies, if any, will be recorded on CRF.

### Outcomes

#### Primary outcome

The primary outcome is the incidence of TLP, which is defined as ≥ 10 erythrocytes/μL of the CSF sample, conforming to the standard definition in hemato-oncology [4–14]. Hospital laboratories will routinely determine the red blood cell count (erythrocytes/ μL) from CSF with flow cytometric methods according to their standard procedures.

The incidence of TLP will be separately calculated for both study arms using Eq. 1.

1 $$\mathrm{Incidence\;of\;TLP}=100\times \frac{\mathrm{Total\;number\;of\;TLPs}}{\mathrm{Total\;number\;of\;all\;procedures}}\mathrm{\%}$$

#### Secondary outcomes

Secondary outcomes are the first puncture success rate and the incidence of PDPH related to the LP procedures in both study arms.

The success of performing the procedure at the first attempt is defined by the following conditions: only one skin penetration with the needle is done, an eligible CSF sample is obtained and sent for the laboratory analysis, and/or the intrathecal therapy is delivered, and the primary provider does not change during the procedure. Multiple needle redirections are allowed provided that the needle tip remains beneath the skin. Multiple stylet removals and reinsertions are also allowed.

The percentage proportions of the first puncture success rate will be separately calculated for both study arms using Eq. 2.

2 $$\mathrm{First\;puncture\;success\;rate}=100\times \frac{\mathrm{Total\;number\;of\;succesful\;procedures\;at\;the\;first\;attempt}}{\mathrm{Total\;number\;of\;all\;procedures}}\mathrm{\%}$$

After each study LP procedure, the local study nurse or investigator will give the patient or parents a diary to be filled for reporting perceived symptoms or complications during the day of the procedure and seven subsequent days. These symptoms and complications include PDPH, headache, nausea, backache, fever, leaking, or inflammation at the puncture site. Conforming to the standard definition, PDPH is a severe headache that worsens in sitting or standing positions, eases after lying down, and occurs within 7 days after the procedure. After the week, the study nurse or investigator will contact the patient or parents and inquire about the diary data.

The incidence of PDPH will be separately calculated for both study arms using Eq. 3.

$$\mathrm{Incidence\;of\;PDPH}=100\times \frac{\mathrm{Total\;number\;of\;procedures\;causing\;PDPH}}{\mathrm{Total\;number\;of\;all\;procedures}}\mathrm{\% }$$

#### Other outcomes

Incidences of other complications than PDPH will be evaluated from the information collected into the abovementioned 1-week symptom diaries and hospital electronic health records. After the 4-week follow-up of each study LP procedure, the local study nurse or investigator will search the patient’s electronic health records for potential symptoms, complications, or adverse events that may be related to the LP procedures or the use of the IQ-Tip system. In case of possibly overlapping 4-week follow-up periods, duplicate events are not recorded. Potential causality between the recorded symptoms, complications, or adverse events and the LP procedure or the use of the IQ-Tip system will be evaluated and documented by the local principal investigator using a 4-level scoring system (not related, possible, probable, and causal). If causality is considered possible, the event is recorded for further analysis.

As appropriate for the time point, data on symptoms, complications, device deficiencies, and adverse events immediately after the study LP procedure, during the 1-week (diary) and 4-week follow-ups (hospital electronic health records), will be gathered and their rates will be reported for both study arms.

From the CSF samples, in addition to the erythrocyte count (# of erythrocytes/μL in CSF) needed for the incidence of TLP, leukocyte count (# of leukocytes/μL) and the existence of blasts (yes/no) will be determined. Basic blood tests (# of erythrocytes, hematocrit, hemoglobin, # of leukocytes, # of thrombocytes, and the existence of blasts) will be performed from the peripheral blood samples according to standard sample processing and flow cytometric procedures of the hospitals. These laboratory data will be reported for both study arms.

The number of attempts needed for a successful LP procedure will be recorded for each study procedure. Congruent with the definition of the first puncture success rate, an attempt denotes any new penetration of the skin. Being successful means that, irrespective of the number of attempts, a CSF sample is eventually obtained and/or the intrathecal therapy is performed as appropriate for the given procedure. The number of failed LP procedures will be registered as well. An LP procedure is considered failed if the physician(s), even after multiple attempts, conclude that the subarachnoid space cannot be verifiably reached, and the procedure remains incomplete with the available personnel or equipment due to any reason. Also, the provider’s decision to use other than the assigned spinal needle due to any reason is considered a failed procedure. These procedural outcomes will be reported for both arms.

#### Performance of the IQ-Tip system

For the study arm A only, the CSF detection sensitivity and false detection rate of the IQ-Tip system will be determined as performance outcomes using the data registered in the patient-specific CRFs. The total numbers of correct alarms (i.e., # of true positive (TP) detections when the CSF detection alarm occurs and CSF flows after the stylet removal), missing alarms (i.e., # of false-negative (FN) detections when the CSF detection alarm do not occur, but CSF flows after the stylet removal), and false alarms (i.e., # of false-positive (FP) detection when the CSF detection alarm occurs but CSF does not flow after the stylet removal) will be recorded.

The CSF detection sensitivity is calculated using Eq. 4.

4 $$\mathrm{CSF\;detection\;sensitivity}=100\times \frac{\mathrm{Total\;number\;of\;TPs}}{\mathrm{Total\;number\;of\;LP\;procedures}}\mathrm{ \%}$$

The false detection rate is calculated using Eq. 5.

5 $$\mathrm{False\;detection\;rate}=100\times \frac{\mathrm{Total\;number\;of\;FPs}}{\mathrm{Total\;number\;of\;LP\;procedures}}\mathrm{ \%}$$

#### Descriptive data

At baseline, patient’s age, height, weight, and sex will be collected for descriptive purposes. The patient’s weight will be categorized into underweight (BMI < 17 kg/m^2^), normal weight (17 ≤ BMI < 25 kg/m^2^), overweight (25 ≤ BMI < 30 kg/m^2^), and obese (BMI ≥ 30 kg/m^2^) by applying the patients’ BMI adjusted for age [30]. The patient’s position during the study LP procedures and the level of anesthesia will be registered. Also, the provider’s experience in performing LP procedures will be requested and divided into three categories (< 10, 11–100, and > 100 LPs performed before the study).

### Data collection and management

The data collection of each study LP procedure will take place at different time points: immediately after performing the procedure, after a week, and after the 4-week follow-up, as described above.

As the participant may undergo up to four study LP procedures during the study, color-coded CRFs will be prepared for each procedure and compiled into the Participant File of the given patient. Pseudonymized copies of the Participant Files will be regularly delivered to the sponsor for data analysis, but no interim analyses are preplanned. However, reported safety data on symptoms, complications, and adverse events will be regularly checked to ensure that safety or vigilance issues requiring further actions will be detected in time.

In case of a device-related serious adverse event with at least a probable link to the use of IQ-Tip system, the local investigator (i.e., the provider of the given LP procedure), the principal investigator, and the sponsor representatives will discuss the incident and decide within 3 days whether the enrolment of participants and the trial need to be suspended. If the suspension is attributed to the medical device, National Competent Authority will be informed.

If the issues underlying the trial suspension cannot be resolved within a reasonable timeframe and potential safety issue persists, the trial may be prematurely terminated upon the decision of the principal investigator and the sponsor. Ethics committee and National Competent Authority will be informed about premature termination. Suspension or premature termination of the trial does not affect the participants’ treatment and care.

During the study LP procedures of the study arm A, the raw bioimpedance measurement data will be collected into the analyzer memory, wherefrom the data will be regularly transferred to a computer and backed-up on an external memory. Participant’s identity cannot be recognized from the raw data, but if deemed necessary, the data can be traced to an individual participant’s identity using the time and date information stored along with the raw bioimpedance data and the original Participant’s File.

Original Participant Files and signed informed consent forms will be archived at the collaborating university hospitals for 15 years.

### Monitoring

The principal investigator (SP) and the sponsor representatives (HS and JK) comprise a team which is responsible for daily conduct and progress of the trial throughout the study parallel with the formal monitoring. The monitoring plan (date 30/09/2022) dictates the principles of how the conduct of the trial at the investigational sites will be audited. The sponsor will be responsible for monitoring.

Besides the site initiation visits before the start and the site close-out visits after the last LP procedure of the study, at least two monitoring visits to each investigational site are planned, the first within about 2 months after the first study procedure and the second one about a few months before the last anticipated procedure.

The focus of the site monitoring and the close-out visits will be on the following issues.

- Informed consent is properly obtained from each participant.
- Processes for individual data protection are obeyed.
- Study LP procedures are performed in the allocated order using the proper spinal needle.
- Investigators are familiar with the guidance about reporting potential adverse events, serious adverse events, and device deficiencies to the sponsor.
- The original CRFs are properly filled and signed.
- The CRF copies delivered to the sponsor match the original CRFs.

Accuracy of data transferred from the symptom diaries and electronic health records to the CRFs will be verified by random sampling of three CRFs filled during the preceding two months. Violations of the protocol will be registered and managed as appropriate.

### Data analysis

Mean, standard deviation (SD), median, interquartile range (IQR), range, and proportions will be used as the main descriptive statistics as appropriate for the data. A cumulative distribution curve will be used as the main descriptive statistics for erythrocyte count data in CSF [14]. This curve illustrates the respective proportions of CSF samples, which denote the incidence of TLP for any given criterion of the erythrocyte count. For the chosen criterion, the incidence of TLP is the vertical distance (in %) from the corresponding curve point up to the 100% point.

**Fig. 2:**
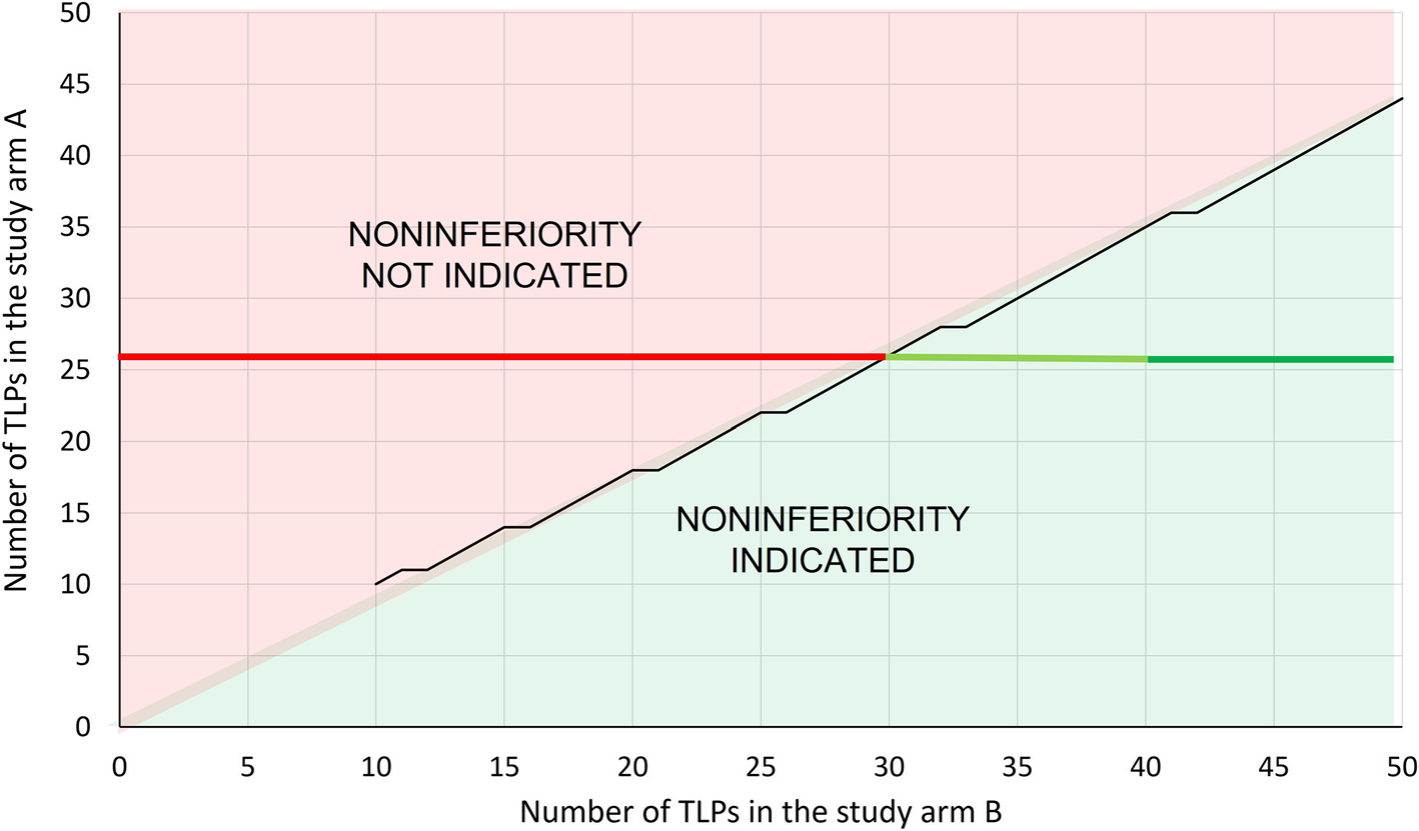
Schematic on the relation between the numbers of traumatic LP procedures (TLP) with the IQ-Tip system and conventional spinal needle required to indicate noninferiority (green area) or not (red area)

The sample size was estimated by an online power calculator for a binary outcome in a non-inferiority trial [31]. For the estimation, it was assumed that the incidence of TLP with the IQ-Tip needle system (study arm A) is 20% and 30% with the conventional spinal needle (study arm B). The values are based on our data on the IQ-Tip system [19] and conventional needle [14] in pediatric hemato-oncology patients, respectively. Considering the 5% noninferiority margin, about 150 LP procedures in both arms will be needed to achieve 90% statistical power to demonstrate noninferiority at the significance level of *p* < 0.05. Assuming a total of 26 TLPs out of 150 procedures in the arm A, corresponding to the 17.3% incidence of TLP in our previous study [19], the total number of TLPs in the arm B should be at least 30 (20%) to demonstrate noninferiority, and at least 40 (26.7%) to demonstrate superiority for the IQ-Tip system. These relationships are illustrated in Fig. 2.

The primary statistical comparison of outcome variables will be based on the true use of the allocated spinal needles in the study LP procedures. Therefore, per-protocol analysis will be employed in the efficacy analysis as recommended for noninferiority trials [32]. The incidences of TLP and PDPH as well as the first puncture success rate between the respective study arms will be compared with the Chi-squared test. The 95% confidence intervals (95% CI) for the difference will be calculated using the exact Clopper-Pearson method. Between-arm comparisons of blood and CSF data, other performance parameters, and incidences of other complications will be performed for descriptive purposes. Definitions of the outcome variables to be used in the efficacy analysis are presented in Table 2 in terms of domain, measurement, metric, aggregation, and time point according to a recommended framework for clinical trials [33, 34]. In case of missing data, data estimation nor imputation will not be performed.

**Table 2:** Definitions of the outcome variables (only those domains pertinent to the efficacy analysis are presented) in terms of domain, measurement, metric, aggregation, and time point

Domain	Specific measurement	Metric	Method of aggregation	Time point
Incidence of TLP	Flow cytometry	Erythrocyte count ≥ 10/µL (yes/no)	Proportion	Each study LP procedure
First puncture success rate	Provider’s self-report on CRF	Occurrence (yes/no)	Proportion	Each study LP procedure
Incidence of PDPH	Symptom diary	Occurrence (yes/no)	Proportion	Within 1 week after each study LP procedure
Peripheral blood analysis	Flow cytometry	Erythrocyte count (cells/µL) Hematocrit (%) Hemoglobin (g/L) Leukocyte count (cells/µL) Thrombocyte count (cells/µL) Existence of blasts (yes/no)	Mean Mean Mean Mean Mean Proportion	Each study LP procedure
CSF analysis	Flow cytometry	Erythrocyte count (cells/µL) Leukocyte count (cells/µL) Existence of blasts (yes/no)	Cumulative distribution curve Mean Proportion	Each study LP procedure
Number of LP attempts	Provider’s self-report on CRF	Number	Median	Each study LP procedure
Number of failed procedures	Provider’s self-report on CRF	Occurrence (yes/no)	Proportion	Each study LP procedure
Incidence of symptoms, complications, and adverse events	Search from hospital electronic health records	Occurrence (yes/no)	Proportion	Within four weeks after each study LP procedure

As an exploratory analysis, logistic regression analysis will be performed to assess factors that may account for the incidence of TLP, defined as a dichotomous variable (yes/no) by the criterion of ≥ 10 erythrocytes/μL. The TLP values will be obtained from the patients’ 2nd, 3rd, and 4th study LP procedures. The following dichotomous variables (yes/no) will be entered to the model as predictors: whether the preceding procedure was performed with a conventional spinal needle, whether the preceding procedure was TLP, whether the consecutive procedures were performed within a week (≤ 7 days) or two (≤ 14 days), whether the patient was obese (BMI ≥ 30 kg/m^2^), whether the provider was experienced with more than 100 previous LP procedures, and whether the thrombocyte count was less than 50,000 platelets/μL [11, 12, 14, 19]. Estimated odds ratios (OR) of the entered predictors will be reported with 95% CIs. The robustness of predictors and related ORs will be verified by performing both backward and forward regression analyses.

Statistical analyses will be done with a concurrent version of IBM SPSS Statistics for Windows (IBM Corp., Armonk, NY, USA).

## Discussion

In Finland, about 50 new pediatric patients with ALL are diagnosed every year, and they all are potentially eligible for the present study. Based on our previous experience [19], these young patients and their parents are interested in participating in clinical studies like the present one. Since this study will be conducted in five university hospitals responsible for the treatment of all childhood cancer patients in Finland, the present study sample will be representative of this patient population. Further, the study LP procedures will be conducted within the routine clinical workflow of pediatric hemato-oncology departments, which enhances the pragmatic value of the study within the actual clinical context. Finally, all the study procedures and data collection methods planned to be used in the present study have already been tried out and found feasible for the purpose in our previous single-arm study [19]. Therefore, the overall risk of failure of the present study is deemed negligible.

Being able to perform an LP procedure is one of the fundamental practical skills of every physician. The LP procedures in the study arm B will be performed with conventional Quincke-type spinal needles, which is congruent with established clinical practice. The procedures in the study arm A, in turn, will be performed with the IQ-Tip system, which presents a novel option designed comparable to the standard LP procedure, the added CSF detection functionality and need for an additional cable and analyzer excluded. In addition to two clinical feasibility studies comprising 69 adult and 40 neonatal and infant LP procedures [25, 26], the system has been used in 152 intrathecal therapy procedures of children and adolescents with ALL [19]. According to the SUS-based usability evaluation, the users in the latter study found the system easy to learn and intuitive to use but some expressed a few concerns about the cable and the reliability of CSF detection alarms. The mean SUS score was 75, which is quite satisfactory, for example, in terms of SUS-based results of graphical user interfaces (mean score 76) or cell phones (mean score 66) [28], or a virtual reality-based biopsy simulator (mean score 75) [35]. Further, the present rating was based on the opinions of 20 different physicians representing a broad range of previous experience in performing LP procedures, from less than 10 to hundreds of procedures. All the above issues corroborate the clinical usability of the system and imply a successful method adoption in the present study as well.

Notwithstanding the present study is a non-inferiority trial by design, we expect that the use of the IQ-Tip system will result in a lower incidence of TLP compared with the conventional spinal needle. This expectation rests on the observed incidences of TLP in our previous clinical study [19] and reference data [14]. The first puncture success rate of the IQ-Tip system was 80%, and when the procedure succeeded at the first attempt, the incidence of TLP was about 11% [19]. If this can be reproduced in the present study, one can at least speculate on that the smaller proportion of traumatic procedures could translate into patients’ better event-free survival in the long term. According to the literature [4, 5, 7, 10, 11], blasts can reside in over 50% of CSF samples drawn from traumatic LP procedures at the beginning of the patients’ intrathecal therapy. Blasts in CSF are known to reduce the probability of event-free survival within the timeframe from 5 to 10 years [4, 5, 7, 10, 11]. This increased risk may concern two to four out of 10 patients with ALL, whose initial LP procedures were traumatic and contaminated by blasts. Based on the electronic health record data from two Finnish university hospitals [14], about 20% of diagnostic LP procedures were traumatic as per the threshold of 10 erythrocytes/μL. This means that approximately 10% of CSF samples of the diagnostic procedures may contain blasts and thus indicate a poorer long-term prognosis. Reflecting the above numbers to 50 new annual cases in Finland, this would mean one to two patients every year who will have a poorer long-term recovery since blasts have contaminated their CSF because of TLP. Speculatively, if we were able to halve the incidence of TLP in pediatric patients with ALL, at least one additional child with ALL (2% of the target population) would survive without CSF-related malignant events in Finland. Considering the new cases at the European level [2], this improvement would mean more than 100 new successfully recovered children. Since the current patient’s treatment protocol of ALL is based on personalized risk assessment [3], avoiding an initial TLP may alleviate the evaluation of personal risk leading to a less intense chemotherapy protocol, which again would reduce the patients’ total burden from intrathecal and systemic chemotherapy [10, 36].

Early stylet removal technique, proposed already in the late 80 s [37], has been associated with a higher success rate of LP procedures [38–40], but not necessarily [41]. A recent large randomized controlled trial of neonates showed that the timing of the stylet removal had no discernible effect on the success of LP procedure [42]. Compared with the traditional “blind” puncture, the fundamental idea of the early stylet removal technique is to verify the correct anatomic location by observing CSF flow immediately after the needle tip is inside the subarachnoid space. This way overshooting the needle and piercing the highly vascularized plexus of the ventral epidural space may be avoided and consequent hemorrhage into the subarachnoid space prevented. However, depending on the CSF pressure, length, and lumen size of the spinal needle, the CSF flow may take a few seconds through the needle cannula before being detected, and the provider may continue to advance the needle. This may particularly happen if the haptic feedback from the perforated dura is somehow perturbed, e.g., because of accumulated scar tissue caused by several previous LP procedures of the patient or different haptic feedback from the needle tip without a stylet. Whereas employing the continuous bioimpedance measurement at the needle tip, the IQ-Tip system can detect CSF in real-time. Instead of waiting for the first drop of CSF, the system immediately gives an alarm when the needle tip has reached the correct location for drawing the CSF sample or injecting the intrathecal therapy. To prevent false-positive CSF detections, the provider needs to be careful that the needle tip does not get out from the subarachnoid space when removing the stylet from the needle. Naturally, the same prudence needs to be exercised when the LP procedure is performed with a conventional spinal needle.

The present study is a part of the mandatory post-market clinical follow-up of the novel IQ-Tip system within the scope of its intended use. As per the regulation MDR (EU 2017/745), manufactures of medical devices are required to conduct clinical evaluation of their products. For high-risk medical devices, including spinal needles because of their contact with the central nervous system, the requirements are stringent and rigorous clinical investigations are required. Serious adverse events or unforeseen risks that would be probably linked to the use of the IQ-Tip system or the device per se are not anticipated, but their existence cannot be ruled out. Therefore, the more accumulated clinical data gathered with the given device without serious adverse events, the higher the certainty of its overall clinical safety. Besides serving the regulatory purpose, the present trial will provide sound scientific evidence on the clinical benefit, performance, and safety of the IQ-Tip system compared with the standard clinical practice of using conventional spinal needles in the LP procedures of pediatric hemato-oncology patients.

### Trial status

The first study procedure was performed on 11 May 2023. The protocol version is 1.1 (date 13 April 2022).

## Data Availability

The trial dataset generated and/or analyzed during the current study will not be publicly available due to ethical and data privacy issues in health care but will be partly available from the corresponding author or sponsor upon reasonable request. The CRF templates for both study arms may be delivered upon reasonable request. The principal investigator and sponsor representatives will have access to the final pseudonymized trial dataset, and they and the local principal investigators (i.e., the research group) will discuss and agree on the authorship, relevant avenues of reporting, and dissemination of the trial results to different target groups. The focus will be on high-quality open-access scientific publications. Professional writers will not be used.
